# Gastrointestinal function and nutritional interventions in septic shock

**DOI:** 10.1097/MCC.0000000000001302

**Published:** 2025-07-09

**Authors:** Kaspar F. Bachmann, Antonella Cotoia, Annika Reintam Blaser

**Affiliations:** a Intensive Care Unit, Department of Acute Medicine, University Hospital Basel, Basel, Switzerland; b Department of Anaesthesiology and Intensive Care, University of Tartu, Tartu, Estonia; c Department of Anaesthesiology and Intensive Care, University Hospital of Foggia, Foggia, Italy; d Department of Intensive Care Medicine, Lucerne Cantonal Hospital, Lucerne, Switzerland

**Keywords:** critical illness, enteral nutrition, gastrointestinal dysfunction, septic shock

## Abstract

**Purpose of review:**

Gastrointestinal (GI) dysfunction significantly impacts patient outcomes in septic shock, complicating clinical management due to its central role in systemic inflammation, barrier integrity, and nutrient assimilation. This review summarizes the evolving understanding of GI dysfunction during septic shock and provides an updated framework for clinical management.

**Recent findings:**

New insights from recent studies focus on individualized nutritional strategies over standardized calorie-driven targets, highlighting risks associated with aggressive enteral nutrition, such as exacerbation of gut ischemia and bowel distension, and microbial dysbiosis. Maintaining splanchnic perfusion, monitoring GI dysfunction with standardized tools, and advancing nutritional support progressively based on patient-specific gastrointestinal tolerance are current strategies. Novel adjunctive therapies targeting gut permeability and microbiome restoration have been proposed, yet robust clinical data remain limited.

**Summary:**

Clinical management should prioritize hemodynamic stabilization and organ support rather than immediately targeting any nutritional goals. Monitoring GI function systematically and tailoring nutritional interventions may prevent complications and support recovery. Future research should validate monitoring tools, refine individual patient assessment, and evaluate novel therapeutic interventions to improve patient-centered outcomes in septic shock.

## INTRODUCTION

Septic shock is characterized by a dysregulated response to infection leading to systemic inflammation, multiorgan dysfunction, and mortality [1]. The gastrointestinal (GI) tract plays a central role in nutrient assimilation, immunological defense, and maintenance of barrier integrity [2^▪^]. Disturbances in GI function are common in septic shock and can contribute to the development of multiple organ dysfunction syndrome (MODS) [3^▪^].

This review summarizes the current evidence on the impact of septic shock on GI function, interorgan interactions from a GI perspective, methods for monitoring GI dysfunction, management strategies for GI dysfunction, and nutritional interventions. 

**Box 1:**
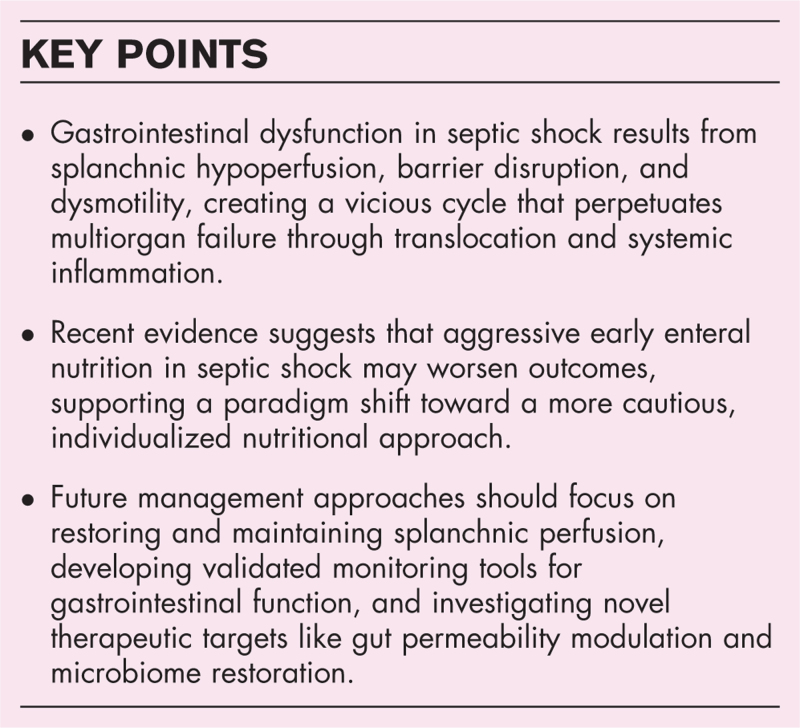
no caption available

## IMPACT OF SEPTIC SHOCK ON GASTROINTESTINAL FUNCTION

A main feature of septic shock is the systemic vasodilation and capillary leak that lead to hypotension [4^▪^], requiring vasopressors that shunt blood away from the splanchnic circulation [5,6]. Experimental models demonstrated that septic conditions reduce intestinal microcirculatory blood flow, triggering mucosal ischemia [7], contributing to cellular energy depletion, mitochondrial dysfunction, and apoptosis of enterocytes. This leads to subsequent barrier disruption. The intestinal barrier comprises a single layer of epithelial cells joined by intercellular junctions that regulate selective permeability and prevent the translocation of bacteria and bacterial products from the gut lumen into the systemic circulation [8]. Both hypoperfusion and the potential ischemia-reperfusion injury lead to the loosening of tight junctions [8]. Sharma and colleagues have shown that alterations in molecular regulators, such as increased levels of noncoding RNAs, impair epithelial renewal and junctional integrity [9^▪^]. Consequently, the compromised barrier permits translocation of bacterial products such as endotoxins (lipopolysaccharide, LPS) into the systemic circulation, exacerbating systemic inflammation and perpetuating a cycle of injury [10^▪▪^].

**FIGURE 1:**
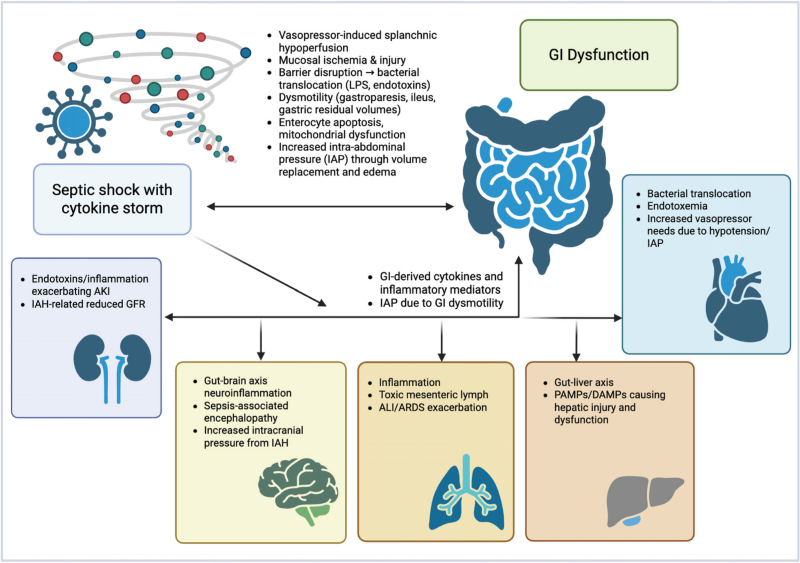
Pathophysiology of gastrointestinal (GI) dysfunction and interorgan interactions in septic shock [88]. Septic shock initiates a systemic inflammatory response (“cytokine storm”), which impairs GI function through vasopressor-induced splanchnic hypoperfusion, mucosal ischemia, barrier disruption, dysmotility, and increased intra-abdominal pressure (IAP). These disturbances lead to bacterial translocation and endotoxemia, exacerbating dysfunction in interconnected organs, including cardiovascular, pulmonary, renal, hepatic, and neurological systems. Each organ system, in turn, contributes to the amplification and perpetuation of septic shock and multiorgan dysfunction syndrome, creating a bidirectional interaction. ALI/ARDS, acute lung injury/acute respiratory distress syndrome; DAMP, damage-associated molecular patterns; GFR, glomerular filtration rate; GI, gastrointestinal; IAH, intra-abdominal hypertension; PAMP, pathogen-associated molecular patterns.

High circulating levels of proinflammatory cytokines, such as tumor necrosis factor-alpha (TNF-α), interleukin (IL)-1β, and IL-6, are central to the host response against septic shock (Fig. 1) [11], but may have deleterious effects on the GI tract by inducing enterocyte apoptosis [12,13]. In septic shock, GI motility's neural and hormonal regulation is disrupted. The coordinated peristaltic movements that propel luminal contents are impaired [14], bowel sounds decreased, potentially indicating autonomic dysfunction induced by sepsis [15^▪▪^,^16^]. Dose- and time-dependent effects of endotoxemia and cytokines on gut motility have been recently shown in animal experiments [17,18]. The dysmotility is associated with delayed gastric emptying and increased gastric residual volumes (GRV), which may be clinically observed as part of enteral feeding intolerance (EFI) [19]. Decreased bowel motility contributes to malabsorption and predisposes the intestine to distension, further compromising the mucosal barrier. The compromised energy status of gut cells exacerbates the susceptibility to ischemic injury and impairs cellular repair mechanisms, thus endangering the integrity of the GI barrier [20^▪^].

## INTERACTIONS BETWEEN GASTROINTESTINAL AND ORGANS DURING SEPTIC SHOCK

These pathophysiologic disturbances are not limited to the gut but contribute to other organ dysfunctions (Fig. 1) [10^▪▪^]. The translocation of bacterial toxins amplifies the systemic inflammatory responses, leading to the development or worsening of MODS [21^▪^]. Thus, GI dysfunction has a bidirectional relationship with other organ dysfunctions/failures, creating a cycle in which worsening gut function promotes further systemic deterioration (Fig. 1) [22].

GI-Cardiovascular: Splanchnic hypoperfusion in septic shock can trigger translocation and systemic inflammation, exacerbating cardiovascular dysfunction [23]. Acute gastrointestinal injury (AGI) in septic shock patients is associated with worse hemodynamic parameters, including higher heart rate, lower mean arterial pressure (MAP), and increased incidence of oliguria [24]. Gut barrier dysfunction during septic shock can lead to impaired cardiac function via endotoxemia [25]. Intra-abdominal hypertension (IAH), occurring due to GI dysfunction, reduces cardiac preload. This can lead to reduced cardiac output and hypotension and may necessitate higher doses of vasopressors to maintain perfusion [26].

GI-Pulmonary: The inflammatory cascade stemming from GI-released bacteria and endotoxins can exacerbate pulmonary dysfunction, leading to acute respiratory distress syndrome (ARDS) [27], worsening pulmonary outcomes [28]. Mesenteric lymph may be a mediator in this process, especially in the case of intra-abdominal sepsis [29]. Conversely, pulmonary dysfunction can exacerbate GI barrier impairment [27,28]. IAH affects pulmonary function by increasing intrathoracic pressure, leading to decreased lung compliance, reduced functional residual capacity, and the development of atelectasis [30].

GI-renal: Gut-derived inflammatory mediators and endotoxins that enter the bloodstream may exacerbate renal injury [31,32]. Acute kidney injury (AKI) leads to the accumulation of uremic toxins and inflammatory mediators, which creates a vicious cycle of organ dysfunction [31,32]. IAH impairs renal perfusion due to direct compression of renal vasculature and increased renal venous pressure. IAH is associated with a higher incidence of AKI, and the severity of IAH correlates with worse renal outcomes and mortality [33,34]. Excessive nutritional support during septic shock may exacerbate kidney injury and increase the necessity of RRT [35^▪▪^].

GI-brain: Bacterial translocation due to increased intestinal permeability can lead to neuroinflammation, contributing to sepsis-associated encephalopathy, driven by inflammatory signals [36]. Animal studies suggest that IAH exacerbates brain dysfunction in sepsis by increasing intracranial pressure, disrupting the blood-brain barrier, and promoting neuroinflammation and neuronal apoptosis [37,38].

GI-liver: Bacterial translocation results in the release of pathogen-associated molecular patterns (PAMPs) and damage-associated molecular patterns (DAMPs) into the liver, contributing to sepsis-induced liver injury [39]. IAH leads to gut microbiota dysbiosis, potentially exacerbating liver injury, and the resulting translocation and systemic inflammation may further impair liver function [40,41].

## GASTROINTESINAL DYSFUNCTION AND ITS MONITORING IN CRITICAL ILLNESS

We did not identify any larger studies assessing GI dysfunction in septic shock. This is explained mainly by difficulties in assessing GI dysfunction in critically ill patients. One small study demonstrated higher and increasing AGI grades during the first 10 days in the ICU in patients with septic shock (*n* = 30) vs. sepsis without shock (*n* = 28) [42]. A recent systematic review showed sepsis and vasoactive drugs being associated with more EFI [43^▪▪^].

The proportion of patients with sepsis in available randomized controlled trials testing different nutritional interventions ranged from 22% to 49% (EPaNIC, PRECISe, EFFORT Protein, PERMIT, TARGET) [44^▪^,^45^^▪▪^]. The proportion of patients with septic shock in nutrition trials, including only patients on vasopressors, was around 60%, with roughly 85% of all patients receiving antibiotics at baseline [46,47]. Nutrirea-2 reported a higher prevalence of vomiting, diarrhea, mesenteric ischemia, and Ogilvie's syndrome in patients receiving full EN vs. full parenteral nutrition (PN) [46]. These symptoms and syndromes, except Ogilvie's syndrome, were also more prevalent in the high (majority achieved with EN) vs. the low dose nutrition group in the Nutrirea-3 trial [47].

That patients receiving higher doses of EN develop GI symptoms more often may appear to be expected [48^▪^,49^▪^]. For years, EFI has been considered an annoying obstacle to providing EN, based on the idea that urgent covering of estimated energy requirements is needed in critically ill patients, and EFI needs to be overcome to provide full-dose EN. However, none of the recent studies have confirmed any benefit of early full nutrition, and no advantages of EN compared to PN were shown when both routes provided a similar dose of energy [44^▪^]. Therefore, EFI may indicate GI dysfunction in response to EN [50^▪^]. Considering GI dysfunction as an organ dysfunction, EN can worsen MODS instead of being an effective treatment strategy. Importantly, it has not been convincingly shown that any interventions currently used to manage EFI (prokinetics, laxatives, postpyloric EN) may improve patient-relevant outcomes [51,52]. Moreover, targeting one GI problem often exacerbates another (e.g., treatment of GI paralysis resulting in diarrhea, or postpyloric feeding in small bowel distension) without any overall improvement in GI function.

The lack of definitions and monitoring tools to measure GI function and dysfunction has suppressed research. In many studies, EFI is not uniformly defined and refers solely to the upper GI tract (gastroparesis) [51]. There is no gold standard to measure GI function, and monitoring of GI dysfunction is largely based on subjective clinical assessment with only a few measurable parameters, such as GRV, volume of diarrhea, and intra-abdominal pressure, that do not apply to all ICU patients [53^▪^]. A recent consensus process defined 13 essential variables that should be documented in clinical studies assessing GI dysfunction and/or EN in critically ill patients [53^▪^]. Consensus definitions for these variables (containing symptoms, signs, clinical entities, and interventions) were also provided and aim to unify reporting in future studies. A prospective multicenter observational study aims to validate a GI dysfunction score [54], allowing GI dysfunction to be assessed as an outcome in future nutrition and GI management studies.

Whereas clinical assessment focuses on GI motility needed to perform nutrient assimilation – just one of the functions - the options for clinical monitoring of other important functions (endocrinological, immune, barrier) are fairly absent. However, they may play a central role in GI dysfunction and its complications and can potentially become therapeutic targets. Endocrine function of the GI tract should ensure adequate digestive and metabolic processes by releasing different hormones produced in enteroendocrine cells [55]. Several enterohormones have been studied in critically ill patients, mainly aiming to identify their potential role in the monitoring of GI function [56], but no biomarker useful in clinical practice has emerged. Association of fibroblast growth factor (FGF-19), a hormone regulating bile acid synthesis, with the development of GI dysfunction in sepsis has been shown [57^▪▪^]. Sepsis and septic shock are frequently linked to lower citrulline levels, potentially reflecting a loss of functional enterocytes crucial for nutrient absorption. Elevated intestinal fatty acid–binding protein (I-FABP) levels have been proposed to signal intestinal mucosal damage. However, data are controversial [42,58,59].

Recent research has suggested that enteroendocrine cells have the ability to sense microbial peptides (gut microbiota) and metabolites, thereby participating in the regulation of immune function and inflammation [60^▪^]. As the gut barrier needs to ensure a balance between tolerance and activation of protection, the gut microbiome is essential for immune system development and function [61^▪^]. The disrupted GI barrier and treatment with antimicrobials, resulting in dysbiosis, lead to a loss of balance between the host and microbiome. Different enterotypes (Bacteroides-dominated and Enterococcus-dominated enterotypes) in critically ill patients with sepsis have been identified [62]. In septic shock patients, the Bacteroides-dominated enterotype was more dominant, and it may be associated with a more severe clinical course and impaired outcome [63,64^▪^].

## POTENTIAL STRATEGIES FOR GASTROINTESTINAL MANAGEMENT IN SEPTIC SHOCK

Overall systemic management of septic shock should aim to preserve the prerequisite for all GI functions – splanchnic circulation – by maintaining euvolemia while minimizing venous congestion. Animal research demonstrated that intestinal microcirculation may be reduced by 75% at a total blood loss of 5% [65^▪▪^]. Medications commonly used in patients with septic shock, such as antimicrobials, vasopressors, and sedatives, unavoidably influence the microbiome and GI motility. Moreover, providing enteral nutrients increases blood flow demand, which may not be fulfilled in shock states and may lead to nonocclusive mesenteric ischemia. Accordingly, the provision of EN in patients with septic shock is potentially harmful. As endogenous energy production is high in the shock state, energy provision may not be needed.

On the other hand, the provision of EN may help to avoid mucosal atrophy [66], preserve the microbiome [67^▪^], and improve immune function [68,69]. Additionally, prolonged enteral starvation may increase the likelihood of GI dysmotility.

Modulation of gut permeability and restoration of the microbiome composition have been recently suggested as potential novel treatment targets [61^▪^,^70^]. Epidermal growth factor, myosin light chain kinase, and caspase inhibitor have been suggested as potential modulators of gut permeability, but no clinical data are available yet. Microbiome-targeted therapies have been more studied [61^▪^]. However, evidence in critically ill and even more so in septic shock patients is limited and controversial regarding pre, pro- and symbiotics, as well as for fecal transplantation. Selective decontamination of the digestive tract is another controversial intervention, whose impact on the microbiome has not been studied. EN as a factor influencing microbiome is generally accepted, but it is unclear how different amounts and formulas influence the microbiome in an individual patient. Too much EN, leading to increased blood flow demand that cannot be met, or giving no enteral nutrients, leading to gut atrophy and dysbiosis, may both be harmful. Available studies rarely assessed any interventions to improve GI function with consequent improvement of patient-relevant outcomes through appropriate management of GI dysfunction as a part of MODS.

## NUTRITIONAL INTERVENTIONS IN SEPSIS AND SEPTIC SHOCK

The timing and dose of EN initiation in critically ill patients play a crucial role in clinical outcomes. ESPEN (European Society for Clinical Nutrition and Metabolism) guidelines recommend starting EN in hemodynamically stable septic ICU patients, including patients on vasopressors or inotropes, given that tissue perfusion targets are reached and maintained under stable or decreasing dose of these medications [71^▪^]. Hemodynamic stabilization may be defined as maintaining a MAP of at least 65 mmHg with fluid resuscitation and vasopressors to optimize tissue perfusion while limiting fluid overload [72]. EN should be started with a low dose and gradually increased based on gastrointestinal tolerance. In patients who do not tolerate full-dose EN during the first week in the ICU, the safety and benefits of initiating PN should be weighed on a case-by-case basis [71^▪^]. Controversially, American guidelines suggest that either low to full doses of EN or PN, but no supplemental PN, can be considered during the first 7–10 days of critical illness, without specific recommendations for septic or septic shock patients [73].

Regarding protein administration, European guidelines suggest that a protein dose of up to 1.3 g/kg/day can be gradually achieved in parallel with the progression to the energy target. In contrast, American guidelines recommend protein intakes from 1.2 to 2.0 g/kg/day from the initiation of nutrition support [71^▪^,^73^].

These controversies in different guidelines are confusing for practitioners and should be improved in future updates. Notably, one of the few changes in the recent update of the ESPEN guidelines was suggesting “reasonable” instead of “all” strategies to “optimize” instead of “maximize” EN, reflecting concerns about too aggressive feeding [71^▪^]. A recent study involving COVID-19 patients, who reached a median maximum caloric goal of 1800 kcal by the 5th day, demonstrated that higher severity scores correlated with increased prevalence of EFI [74]. This confirms earlier findings that EFI is associated with the severity of illness. A shift in paradigm would mean that instead of aiming for full EN by maximum measures and initiating supplemental PN, acceptance of staying below an estimated target may be appropriate in most severely ill patients [44^▪^,71^▪^]. However, monitoring of underfeeding is currently not possible in the ICU, and PN in ICU patients is not uniformly defined [75^▪^]. Careful management of nonnutritional calories from propofol and dextrose infusions is essential due to their significant contribution to early caloric intake [76^▪^].

In a recent review, Patel and colleagues summarize evidence against early and aggressive EN in patients with septic shock, advocating an individualized approach [2^▪^]. However, methods to monitor the metabolic status of patients to make individual decisions are currently unavailable.

Although a comprehensive malnutrition assessment may not be feasible during the early stage of sepsis or septic shock due to factors like fluid shifts and clinical instability, malnutrition risk screening tools, such as the GLIM score, can still be valuable [77^▪▪^]. Malnourishment at ICU admission and the development of malnutrition during ICU stay should be clearly distinguished.

## NOVEL OPTIONS FOR MONITORING AND TREATMENT

Supporting gut integrity/barrier function would be desirable. Difficulties in measuring this function of the GI tract complicate testing of potential treatment strategies. Experimental therapies may guide the development of novel therapies. Radix Sanguisorbae (RS, Diyu) may improve the intestinal barrier function by inhibiting ferroptosis in septic rat models, evidenced by decreased intestinal permeability (reduced Evans blue extravasation), restored tight junction protein expression (ZO-1), improved intestinal villi structure (observed via transmission electron microscopy), and reduced mesenteric vessel leakage [78^▪^]. Subcutaneous application of geranylgeranylacetone protected against LPS-induced intestinal permeability by upregulating heat shock protein 70 and inhibiting NF-κB, reducing both inflammatory cytokine production and oxidative stress [79]. Lipid mediators derived from omega-3 fatty acids (eicosapentaenoic acid and docosahexaenoic acid) may play a role in resolving inflammation [80^▪^,^81^].

Alterations in electrolyte levels, particularly abnormal blood phosphate concentration, are common in critically ill patients and may reflect the response to nutrition [35^▪▪^,82^▪^]. An ongoing large multicenter study investigating the prevalence of blood phosphate abnormalities during the first week of ICU will also allow insight into patients with sepsis [83]. Micronutrients such as vitamins, selenium, zinc, and copper are crucial for immune modulation and antioxidant defense [84]. High-dose micronutrients (above nutritional doses) are not recommended [71^▪^], and no specific guidance in patients with sepsis or septic shock exists. An RCT in non-ICU patients suggests that nutritional intervention, including both macro- and micronutrients, may improve outcomes [85]. However, patients with elevated CRP did not seem to profit from this intervention [86]. Even if energy targets are not known in patients with septic shock, discrepancy between the targets and actual administration needs to be acknowledged and monitored, while recent technological advances may be helpful in monitoring and compensating for diagnostic-related interruptions [87^▪▪^].

## CONCLUSION

**FIGURE 2:**
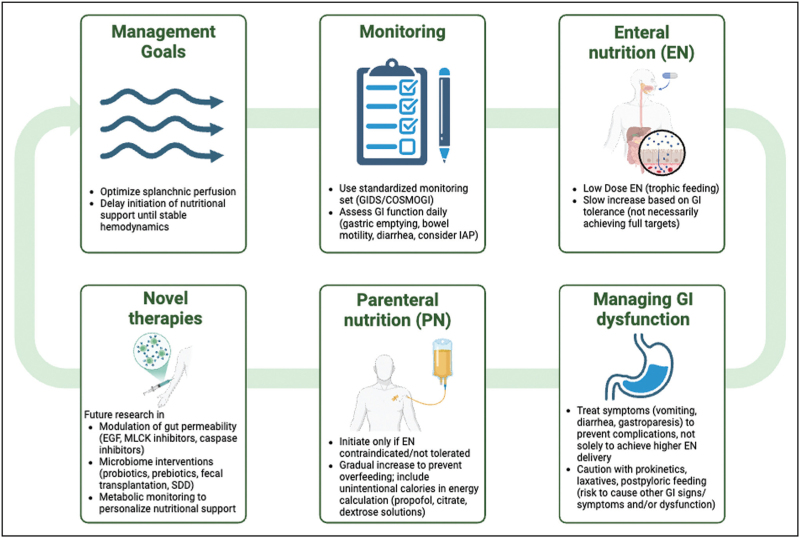
Clinical management algorithm for gastrointestinal (GI) dysfunction and nutritional support in septic shock [89]. Management begins with optimizing splanchnic perfusion and delaying nutritional support until hemodynamic stability is achieved. Systematic monitoring using standardized tools (e.g. GIDS/COSMOGI) to assess GI function guides initiation and gradual advancement of enteral nutrition (EN) based on GI tolerance [53^▪^]. GI dysfunction symptoms should be carefully managed to prevent complications rather than solely to increase EN delivery. If EN is contraindicated or insufficient, parenteral nutrition (PN) should be cautiously initiated to avoid complications of overfeeding. Emerging therapeutic strategies include modulation of gut permeability and microbiome-targeted interventions, representing potential future developments. EGF, epidermal growth factor; GI, gastrointestinal; GRV, gastric residual volume; IAP, intra-abdominal pressure; MLCK, myosin light chain kinase; SDD, selective decontamination of the digestive tract.

GI dysfunction complicates clinical management in septic shock, given its bidirectional relationship with systemic inflammation and multiorgan dysfunction syndrome (MODS). Effective management requires a nuanced approach that prioritizes restoration and maintenance of adequate splanchnic perfusion, careful monitoring of GI function, and thoughtful nutritional strategies. Rather than pursuing maximal nutritional targets, recent evidence advocates balancing nutritional support against potential risks such as exacerbation of gut ischemia, dysmotility, and microbial dysbiosis. Based on the current understanding, we propose a structured clinical pathway focusing initially on hemodynamic stabilization and optimization of gut perfusion before gradually initiating nutritional interventions under careful monitoring of GI dysfunction (Fig. 2). Adjunctive therapies addressing gut permeability and microbiome composition represent promising future directions.

## Acknowledgements

*None.*

### Financial support and sponsorship

*None.*

### Conflicts of interest

*A.R.B. is holding a grant from the Estonian Research Council (PRG1255). K.F.B. and A.C. do not have any conflicts of interest to declare.*

## REFERENCES AND RECOMMENDED READING

Papers of particular interest, published within the annual period of review, have been highlighted as:

▪ of special interest
▪▪ of outstanding interest

## References

[1] Bauer M Gerlach H Vogelmann T. Mortality in sepsis and septic shock in Europe, North America and Australia between 2009 and 2019 – results from a systematic review and meta-analysis. Crit Care 2020; 24:239. 32430052 10.1186/s13054-020-02950-2 PMC7236499

[2▪] Patel JJ Lopez-Delgado JC Stoppe C McClave SA. Enteral nutrition in septic shock: a call for a paradigm shift. Curr Opin Crit Care 2024; 30:165–171. 38441124 10.1097/MCC.0000000000001134

[3▪] Borges A Bento L. Organ crosstalk and dysfunction in sepsis. Ann Intensive Care 2024; 14:147. 39298039 10.1186/s13613-024-01377-0 PMC11413314

[4▪] McMullan RR McAuley DF O’Kane CM Silversides JA. Vascular leak in sepsis: physiological basis and potential therapeutic advances. Critical Care 2024; 28:97. 38521954 10.1186/s13054-024-04875-6 PMC10961003

[5] Liu J Zheng M Zhao X. Effects of vasoactive drugs on hepatic and intestinal circulation and intestinal barrier in patients with septic shock. J Investig Med 2021; 69:833–837. 10.1136/jim-2020-001685 33441480

[6] Premachandra A Heming N. Acute management of sepsis beyond 24 hours. 10.1055/s-0044-1787991 38968962

[7] Seilitz J Grafver I Kiszakiewicz L. A randomized porcine study in low cardiac output of vasoactive and inotropic drug effects on the gastrointestinal tract. Shock 2021; 56:308–317. 33443363 10.1097/SHK.0000000000001726 PMC8529897

[8] Obermuller B Frisina N Meischel M. Examination of intestinal ultrastructure, bowel wall apoptosis and tight junctions in the early phase of sepsis. Sci Rep 2020; 10:11507. 32661347 10.1038/s41598-020-68109-9 PMC7359326

[9▪] Sharma S Xiao L Chung HK. Noncoding vault RNA1-1 impairs intestinal epithelial renewal and barrier function by interacting with CUG-binding protein 1. Cell Mol Gastroenterol Hepatol 2025; 19:101410. 39349247 10.1016/j.jcmgh.2024.101410 PMC11612821

[10▪▪] Magnan C Lancry T Salipante F. Role of gut microbiota and bacterial translocation in acute intestinal injury and mortality in patients admitted in ICU for septic shock. Front Cell Infect Microbiol 2023; 13:1330900. 38179421 10.3389/fcimb.2023.1330900 PMC10765587

[11] Das UN. Infection, inflammation, and immunity in sepsis. Biomolecules 2023; 13:1332. 37759732 10.3390/biom13091332 PMC10526286

[12] Ruder B Atreya R Becker C. Tumour necrosis factor alpha in intestinal homeostasis and gut related diseases. Int J Mol Sci 2019; 20:E1887. 10.3390/ijms20081887 PMC6515381 30995806

[13] Droessler L Cornelius V Markov AG Amasheh S. Tumor necrosis factor alpha effects on the porcine intestinal epithelial barrier include enhanced expression of TNF receptor 1. Int J Mol Sci 2021; 22:8746. 34445450 10.3390/ijms22168746 PMC8395858

[14] Overhaus M Togel S Pezzone MA Bauer AJ. Mechanisms of polymicrobial sepsis-induced ileus. Am J Physiol Gastrointest Liver Physiol 2004; 287:G685–G694. 15331356 10.1152/ajpgi.00359.2003

[15▪▪] Castro M Valero MS Lopez-Tofino Y. Radiographic and histopathological study of gastrointestinal dysmotility in lipopolysaccharide-induced sepsis in the rat. Neurogastroenterol Motil 2023; 35:e14639. 37417393 10.1111/nmo.14639

[16] Greis C Rasuly Z Janosi RA. Intestinal T lymphocyte homing is associated with gastric emptying and epithelial barrier function in critically ill: a prospective observational study. Crit Care 2017; 21:70. 28327177 10.1186/s13054-017-1654-9 PMC5361812

[17] Ohama T Hori M Momotani E. Intestinal inflammation downregulates smooth muscle CPI-17 through induction of TNF-alpha and causes motility disorders. Am J Physiol Gastrointest Liver Physiol 2007; 292:G1429–G1438. 17307724 10.1152/ajpgi.00315.2006

[18] Pazdrak K Shi XZ Sarna SK. TNFalpha suppresses human colonic circular smooth muscle cell contractility by SP1- and NF-kappaB-mediated induction of ICAM-1. Gastroenterology 2004; 127:1096–1109. 15480988 10.1053/j.gastro.2004.07.008

[19] Heyland DK Ortiz A Stoppe C. Incidence, risk factors, and clinical consequence of enteral feeding intolerance in the mechanically ventilated critically ill: an analysis of a multicenter, multiyear database. Crit Care Med 2021; 49:49–59. 33148950 10.1097/CCM.0000000000004712

[20▪] Chen S Shen C Zeng X. Energy metabolism and the intestinal barrier: implications for understanding and managing intestinal diseases. Front Microbiol 2025; 16:1515364. 39959156 10.3389/fmicb.2025.1515364 PMC11826063

[21▪] Soranno DE Coopersmith CM Brinkworth JF. A review of gut failure as a cause and consequence of critical illness. Crit Care 2025; 29:91. 40011975 10.1186/s13054-025-05309-7 PMC11866815

[22] Meng M Klingensmith NJ Coopersmith CM. New insights into the gut as the driver of critical illness and organ failure. Curr Opin Crit Care 2017; 23:143–148. 28092310 10.1097/MCC.0000000000000386 PMC5373099

[23] Tamion F Richard V Sauger F. Gastric mucosal acidosis and cytokine release in patients with septic shock. Crit Care Med 2003; 31:2137–2143. 12973171 10.1097/01.CCM.0000079600.49048.28

[24] Klanovicz TM Franzosi OS Nunes DSL. Acute gastrointestinal failure is associated with worse hemodynamic and perfusion parameters over 48 h after admission in patients with septic shock: retrospective cohort study. Nutr Clin Pract 2023; 38:617–627. 36351616 10.1002/ncp.10928

[25] Nguyen M Gautier T Masson D. Endotoxemia in acute heart failure and cardiogenic shock: evidence, mechanisms and therapeutic options. J Clin Med 2023; 12:2579. 37048662 10.3390/jcm12072579 PMC10094881

[26] Regueira T Bruhn A Hasbun P. Intra-abdominal hypertension: incidence and association with organ dysfunction during early septic shock. J Crit Care 2008; 23:461–467. 19056007 10.1016/j.jcrc.2007.12.013

[27] Zhou X Liao Y. Gut-lung crosstalk in sepsis-induced acute lung injury. Front Microbiol 2021; 12:779620. 35003009 10.3389/fmicb.2021.779620 PMC8733643

[28] Nath S Kitsios GD Bos LDJ. Gut-lung crosstalk during critical illness. Curr Opin Crit Care 2023; 29:130–137. 36762684 10.1097/MCC.0000000000001015

[29] Liu Y Chen C Sun Q. Mesenteric lymph duct drainage attenuates lung inflammatory injury and inhibits endothelial cell apoptosis in septic rats. Biomed Res Int 2020; 2020:3049302. 33145344 10.1155/2020/3049302 PMC7596461

[30] Tonetti T Cavalli I Ranieri VM Mascia L. Respiratory consequences of intra-abdominal hypertension. Minerva Anestesiol 2020; 86:877–883. 32368883 10.23736/S0375-9393.20.14325-6

[31] Xu Y Kong X Zhu Y. Contribution of gut microbiota toward renal function in sepsis. Front Microbiol 2022; 13:985283. 36147845 10.3389/fmicb.2022.985283 PMC9486003

[32] Zhang J Ankawi G Sun J. Gut-kidney crosstalk in septic acute kidney injury. Crit Care 2018; 22:117. 29724256 10.1186/s13054-018-2040-y PMC5934860

[33] Suphatheerawatr N Jaturapisanukul S Prommool S. Intra-abdominal hypertension among medical septic patients associated with worsening kidney outcomes (IAH-WK study). Medicine (Baltimore) 2023; 102:e32807. 36705348 10.1097/MD.0000000000032807 PMC9875967

[34] Bachmann KF Regli A Mandul M. Impact of intraabdominal hypertension on kidney failure in critically ill patients: a posthoc database analysis. J Crit Care 2022; 71:154078. 35738182 10.1016/j.jcrc.2022.154078

[35▪▪] Lauwers C Langouche L Wouters PJ. Early phosphate changes as potential indicator of unreadiness for artificial feeding: a secondary analysis of the EPaNIC RCT. Crit Care 2025; 29:48. 39875953 10.1186/s13054-025-05273-2 PMC11773907

[36] Morris DC Zhang ZG Jaehne AK. Exosomal mechanisms of cardiac and brain dysfunction in sepsis. Shock 2023; 59:173–179. 36731014 10.1097/SHK.0000000000002015

[37] He YJ Xu H Fu YJ. Intraperitoneal hypertension, a novel risk factor for sepsis-associated encephalopathy in sepsis mice. Sci Rep 2018; 8:8173. 29802336 10.1038/s41598-018-26500-7 PMC5970176

[38] Youssef AM Hamidian Jahromi A Vijay CG. Intra-abdominal hypertension causes reversible blood-brain barrier disruption. J Trauma Acute Care Surg 2012; 72:183–188. 22002620 10.1097/TA.0b013e31822a3254

[39] Zhang X Liu H Hashimoto K. The gut-liver axis in sepsis: interaction mechanisms and therapeutic potential. Crit Care 2022; 26:213. 35831877 10.1186/s13054-022-04090-1 PMC9277879

[40] Sun J Zhang J Wang X. Gut-liver crosstalk in sepsis-induced liver injury. Crit Care 2020; 24:614. 33076940 10.1186/s13054-020-03327-1 PMC7574296

[41] Zhao Z Guo Z Yin Z Front Physiol 2021; 12:790182. 34955896 10.3389/fphys.2021.790182 PMC8703017

[42] Tyszko M Lemanska-Perek A Smiechowicz J. Citrulline, intestinal fatty acid-binding protein and the acute gastrointestinal injury score as predictors of gastrointestinal failure in patients with sepsis and septic shock. Nutrients 2023; 15:2100. 37432225 10.3390/nu15092100 PMC10180779

[43▪▪] Wang S He Y Yi J Sha L. Risk factors for enteral feeding intolerance in critically ill patients: an updated systematic review and meta-analysis. BMC Gastroenterol 2025; 25:233. 40200147 10.1186/s12876-025-03837-8 PMC11980324

[44▪] de Man AME Gunst J Reintam Blaser A. Nutrition in the intensive care unit: from the acute phase to beyond. Intensive Care Med 2024; 50:1035–1048. 38771368 10.1007/s00134-024-07458-9 PMC11245425

[45▪▪] Bels JLM Thiessen S van Gassel RJJ. Effect of high versus standard protein provision on functional recovery in people with critical illness (PRECISe): an investigator-initiated, double-blinded, multicentre, parallel-group, randomised controlled trial in Belgium and the Netherlands. Lancet 2024; 404:659–669. 39153816 10.1016/S0140-6736(24)01304-7

[46] Reignier J Boisramé-Helms J Brisard L. Enteral versus parenteral early nutrition in ventilated adults with shock: a randomised, controlled, multicentre, open-label, parallel-group study (NUTRIREA-2). Lancet 2018; 391:133–143. 29128300 10.1016/S0140-6736(17)32146-3

[47] Reignier J Plantefeve G Mira JP. Low versus standard calorie and protein feeding in ventilated adults with shock: a randomised, controlled, multicentre, open-label, parallel-group trial (NUTRIREA-3). Lancet Respir Med 2023; 11:602–612. 36958363 10.1016/S2213-2600(23)00092-9

[48▪] Feng LF Li XW Zhu XQ Jin LN. Advances in management strategies for enteral nutrition-related gastric retention in adult patients with nasogastric tubes. World J Gastrointest Surg 2025; 17:101751. 40162381 10.4240/wjgs.v17.i3.101751 PMC11948141

[49▪] Crone V M⊘ller MH Alhazzani W. Preferences on the use of prokinetic agents in adult intensive care unit patients-an international survey. Acta Anaesthesiol Scand 2025; 69:e70045. 40275492 10.1111/aas.70045 PMC12022387

[50▪] Berger MM Reintam Blaser A Raphaeli O Singer P. Early feeding in critical care - where are we now? Crit Care Clin 2025; 41:213–231. 40021276 10.1016/j.ccc.2024.09.002

[51] Crone V M⊘ller MH Baekgaard ES. Use of prokinetic agents in hospitalised adult patients: a scoping review. Acta Anaesthesiol Scand 2023; 67:588–598. 36847067 10.1111/aas.14222

[52] Li L Huang J. Nasogastric tube versus postpyloric tube feeding for critical illness: a systematic review and meta-analysis. Asia Pac J Clin Nutr 2024; 33:283–297. 38965718 10.6133/apjcn.202409_33(3).0001 PMC11389815

[53▪] Bachmann KF Jenkins B Asrani V. Core outcome set of daily monitoring of gastrointestinal function in adult critically ill patients: a modified Delphi consensus process (COSMOGI). Crit Care 2024; 28:420. 39695807 10.1186/s13054-024-05192-8 PMC11654350

[54] Kouw IWK Melchers M Mändul M. Prospective multicenter study to validate the gastrointestinal dysfunction score (GIDS) in intensive care patients: study protocol for Part A of the international GUTPHOS study. Clin Nutr ESPEN 2024; 63:702–708. 39069258 10.1016/j.clnesp.2024.07.023

[55] Bany Bakar R Reimann F Gribble FM. The intestine as an endocrine organ and the role of gut hormones in metabolic regulation. Nat Rev Gastroenterol Hepatol 2023; 20:784–796. 37626258 10.1038/s41575-023-00830-y

[56] Reintam Blaser A Preiser JC Fruhwald S. Gastrointestinal dysfunction in the critically ill: a systematic scoping review and research agenda proposed by the section of metabolism, endocrinology and nutrition of the European Society of Intensive Care Medicine. Crit Care 2020; 24:224. 32414423 10.1186/s13054-020-02889-4 PMC7226709

[57▪▪] Guan L Wang F Chen J. Clinical value of fibroblast growth factor 19 in predicting gastrointestinal dysfunction in patients with sepsis. Front Nutr 2024; 11:1442203. 39296513 10.3389/fnut.2024.1442203 PMC11408290

[58] Reintam Blaser A Starkopf J Björck M. Diagnostic accuracy of biomarkers to detect acute mesenteric ischaemia in adult patients: a systematic review and meta-analysis. World J Emerg Surg 2023; 18:44. 37658356 10.1186/s13017-023-00512-9 PMC10474684

[59] Nuzzo A Guedj K Curac S. Accuracy of citrulline, I-FABP and D-lactate in the diagnosis of acute mesenteric ischemia. Sci Rep 2021; 11:18929. 34556697 10.1038/s41598-021-98012-w PMC8460675

[60▪] Philpott JD Rodriguez Hovnanian KM Stefater-Richards M. The enteroendocrine axis and its effect on gastrointestinal function, nutrition, and inflammation. Curr Opin Crit Care 2024; 30:290–297. 38872371 10.1097/MCC.0000000000001175 PMC11295110

[61▪] Oami T Shimazui T Yumoto T. Gut integrity in intensive care: alterations in host permeability and the microbiome as potential therapeutic targets. J Intensive Care 2025; 13:16. 40098052 10.1186/s40560-025-00786-y PMC11916345

[62] Liu W Cheng M Li J. Classification of the gut microbiota of patients in intensive care units during development of sepsis and septic shock. Genomics Proteomics Bioinformatics 2020; 18:696–707. 33607294 10.1016/j.gpb.2020.06.011 PMC8377022

[63] Sun S Wang D Dong D. Altered intestinal microbiome and metabolome correspond to the clinical outcome of sepsis. Crit Care 2023; 27:127. 36978107 10.1186/s13054-023-04412-x PMC10044080

[64▪] Shang W Zhang S Qian H. Gut microbiota and sepsis and sepsis-related death: a Mendelian randomization investigation. Front Immunol 2024; 15:1266230. 38361921 10.3389/fimmu.2024.1266230 PMC10867964

[65▪▪] Davies S Jian Z Hatib F. Detection of hypovolaemia by the hypotension prediction index is associated with gastrointestinal microcirculation dysfunction in a porcine model of haemorrhage. Shock 2025; 64:91. 40132803 10.1097/SHK.0000000000002578

[66] Hu Q Ren H Hong Z. Early enteral nutrition preserves intestinal barrier function through reducing the formation of neutrophil extracellular traps (NETs) in critically ill surgical patients. Oxid Med Cell Longev 2020; 2020:8815655. 33294125 10.1155/2020/8815655 PMC7700037

[67▪] Lee S Wischmeyer PE Mintz CD Serbanescu MA. Recent insights into the evolving role of the gut microbiome in critical care. Crit Care Clin 2025; 41:379–396. 40021286 10.1016/j.ccc.2024.11.002 PMC12497176

[68] Covello C Becherucci G Di Vincenzo F. Parenteral nutrition, inflammatory bowel disease, and gut barrier: an intricate plot. Nutrients 2024; 16:2288. 39064731 10.3390/nu16142288 PMC11279609

[69] McClave SA. Can feeding strategies alter immune signaling and gut sepsis in critical illness? JPEN J Parenter Enteral Nutr 2021; 45 (S2):66–73. 34477220 10.1002/jpen.2260

[70] Oami T Yamamoto A Ishida S. Critical care nutrition from a metabolic point of view: a narrative review. Nutrients 2025; 17:1352. 40284216 10.3390/nu17081352 PMC12029973

[71▪] Singer P Blaser AR Berger MM. ESPEN practical and partially revised guideline: clinical nutrition in the intensive care unit. Clin Nutr 2023; 42:1671–1689. 37517372 10.1016/j.clnu.2023.07.011

[72] Guarino M Perna B Cesaro AE. 2023 Update on sepsis and septic shock in adult patients: management in the emergency department. J Clin Med 2023; 12:3188. 37176628 10.3390/jcm12093188 PMC10179263

[73] Compher C Bingham AL McCall M. Guidelines for the provision of nutrition support therapy in the adult critically ill patient: the American Society for Parenteral and Enteral Nutrition. JPEN J Parenter Enteral Nutr 2022; 46:12–41. 34784064 10.1002/jpen.2267

[74] Casas-Jaramillo F Polania-Sandoval CA Perez Rivera CJ. Nutritional and metabolic support in critically-ill patients with COVID-19 disease: a multicenter cohort study. Clin Nutr Open Sci 2023; 52:25–33.

[75▪] Reintam Blaser A Cotoia A Berger MM. How to define parenteral nutrition. Crit Care 2024; 28:372. 39563392 10.1186/s13054-024-05153-1 PMC11577738

[76▪] Popoff B Occhiali E Demailly Z. Evaluation of nonnutritional calories in intensive care patients with acute respiratory distress syndrome due to coronavirus disease-19: a retrospective observational study. Clin Nutr Open Sci 2024; 53:44–56.

[77▪▪] Compher CW Fukushima R Correia M. Recognizing malnutrition in adults with critical illness: guidance statements from the Global Leadership Initiative on Malnutrition. JPEN J Parenter Enteral Nutr 2025; 49:202–208. 10.1002/jpen.2748 PMC12053144 40162679

[78▪] Liu YY Bao DQ Zhang ZS. Radix sanguisorbae improves intestinal barrier in septic rats via HIF-1 alpha/HO-1/Fe(2+) axis. Chin J Integr Med 2024; 30:1101–1112. 38212494 10.1007/s11655-023-3550-2

[79] Liu X Liu Y Su X. Geranylgeranylacetone mitigates sepsis-associated intestinal injury through CHIP-dependent antiinflammation and antioxidative effect. Int Immunopharmacol 2024; 135:112263. 38788444 10.1016/j.intimp.2024.112263

[80▪] Martindale RG Calder PC Cogle SV. Lipids in parenteral nutrition – expert consensus statements: translating guidelines into clinical practice. Clin Nutr Open Sci 2025; 60:50–65.

[81] Heyland D, Lee Z, Lew C, *et al*. Composition of parenteral nutrition: type of lipids. Crit Care Nutr. 2022. https://www.criticalcarenutrition.com/docs/SOE_PNLipidType_22Jan2022.pdf [Accessed 23 May 2025].

[82▪] Bachmann KF Hess B Koitmäe M. Electrolyte disorders in the critically ill: a retrospective analysis. Sci Rep 2025; 15:13943. 40263430 10.1038/s41598-025-98677-7 PMC12015444

[83] Melchers M Kouw IWK Arabi YM. Prospective multicenter study to describe the prevalence, outcomes, and management of phosphate disorders in intensive care patients: study protocol for part B of the international GUTPHOS study. Clin Nutr ESPEN 2024; 63:681–687. 39069259 10.1016/j.clnesp.2024.07.024

[84] de Man AME Stoppe C Koekkoek K. What do we know about micronutrients in critically ill patients? A narrative review. JPEN J Parenter Enteral Nutr 2025; 49:33–58. 39555865 10.1002/jpen.2700 PMC11717498

[85] Schuetz P Fehr R Baechli V. Individualised nutritional support in medical inpatients at nutritional risk: a randomised clinical trial. Lancet 2019; 393:2312–2321. 31030981 10.1016/S0140-6736(18)32776-4

[86] Merker M Felder M Gueissaz L. Association of baseline inflammation with effectiveness of nutritional support among patients with disease-related malnutrition: a secondary analysis of a randomized clinical Trial. JAMA Netw Open 2020; 3:e200663. 32154887 10.1001/jamanetworkopen.2020.0663 PMC7064875

[87▪▪] Kagan I Robinson E Itshaki MH Singer P. Interruptions in administration of enteral feeding and automatic compensation: a post hoc analysis of the smART+ study. Clin Nutr Open Sci 2025; 61:62–69.

[88] Created in BioRender. Bachmann, K. 2025. https://BioRender.com/2gej3n2.

[89] Created in BioRender. Bachmann, K. 2025. https://BioRender.com/r45xl9x.

